# Effects of Multidrug Resistant Tuberculosis Treatment on Patients’ Health Related Quality of Life: Results from a Follow Up Study

**DOI:** 10.1371/journal.pone.0159560

**Published:** 2016-07-28

**Authors:** Nafees Ahmad, Arshad Javaid, Syed Azhar Syed Sulaiman, Anila Basit, Afsar Khan Afridi, Ammar Ali Saleh Jaber, Amer Hayat Khan

**Affiliations:** 1 Discipline of Clinical Pharmacy, School of Pharmaceutical Sciences, Universiti Sains Malaysia, Pulau Pinang, Malaysia; 2 Department of Pulmonology, Postgraduate Medical Institute Peshawar, Peshawar, Pakistan; 3 PMDT, Lady Reading Hospital Peshawar, Peshawar, Pakistan; Indian Institute of Technology Delhi, INDIA

## Abstract

**Background:**

At present, within the management of multidrug resistant tuberculosis (MDR-TB) much attention is being paid to the traditional microbiological and clinical indicators. Evaluation of the impact of MDR-TB treatment on patients’ Health Related Quality of Life (HRQoL) has remained a neglected area.

**Objective:**

To evaluate the impact of MDR-TB treatment on patients HRQoL, and determine the predictors of variability in HRQoL along the course of treatment

**Methods:**

A prospective follow up study was conducted at the programmatic management unit for drug resistant TB of Lady Reading Hospital Peshawar. Culture confirmed eligible MDR-TB patients were asked to self complete SF-36v2 at the baseline visit, and subsequently after the completion of 12 months of treatment and at the end of treatment. A score of <47 norm-based scoring (NBS) points on component summary measures and health domain scales was considered indicative of function impairment. General linear model repeated measures ANOVA was used examine the change and predictors of change in physical component summary (PCS) and mental component summary (MCS) scores over the time.

**Results:**

A total of 68 out of enrolled 81 eligible MDR-TB patients completed SF-36v2 questionnaire at the three time points. Patients’ mean PCS scores at the three time points were, 38.2±4.7, 38.6±4.4 and 42.2±5.2 respectively, and mean MCS were 33.7±7.0, 35.5±6.9 and 40.0±6.9 respectively. Length of sickness prior to the diagnosis of MDR-TB was predictive of difference in PCS scores (F = 4.988, Df = 1, 66), whereas patients’ gender (F = 5.638, Df = 1, 66) and length of sickness prior to the diagnosis of MDR-TB (F = 4.400, Df = 1, 66) were predictive of difference in MCS scores.

**Conclusion:**

Despite the positive impact of MDR-TB treatment on patients' HRQoL, the scores on component summary measures suggested compromised physical and mental health even at the end of treatment. A large multicenter study is suggested to confirm the present findings.

## Introduction

Health Related Quality of Life (HRQoL) is defined as “the extent to which patient’s subjective perception of physical, mental and social wellbeing are affected on a day to day basis by a disease and its treatment(s)”[1]. It is known that patients with chronic diseases, in addition to pure physical health also place high value on their mental and social wellbeing [2]. As a result, evaluation of HRQoL has become an important health outcome and an area of concern for policy makers, health care professionals and researchers. Multidrug resistant tuberculosis (MDR-TB) defined as “TB caused by strain of *Mycobacterium tuberculosis* (MTB) resistant to both isoniazid (INH) and rifampicin (RMP)” is a chronic, debilitating disease of prolonged chemotherapy (≥ 20 months) with a severe regimen of potentially toxic and less potent second-line anti-TB drugs (SLD) [3–4]. In a recently conducted qualitative study in Mumbai, India, MDR-TB patients and their family members described the disease as “the worst of the worst illnesses” and its treatment “worse than the disease itself” [5].

However, at present within the management MDR-TB, much attention is being paid to the traditional microbiological and clinical indicators. Its impact on patients’ HRQoL has remained a neglected area. The review of English language literature revealed very few studies which have evaluated the HRQoL of MDR-TB patients. These studies have done it either cross-sectionally [6–8] or included smaller subsamples of MDR-TB patients among the drug susceptible TB population [9]. Literature review could not identify a single longitudinal study which has evaluated the impact of MDR-TB treatment on patients HRQoL. The authors of WHO’s progress report demarking goals for 2015 in the treatment of MDR-TB, note “there is a dearth of literature about anti-TB drug-induced mortality, morbidity and loss in quality of life, particularly in low-resource settings” [10]. Thus, present study was conducted with the aim to evaluate the impact of MDR-TB, and its treatment on patients’ HRQoL. This study will help in filling the gap of patient reported outcomes among MDR-TB patients, and providing the much needed data regarding the impact of MDR-TB treatment on patients’ HRQoL.

## Materials and Methods

### Study settings, design and population

This was a prospective follow-up study conducted at the programmatic management unit for drug resistant TB of Lady Reading Hospital Peshawar, Pakistan. All eligible (≥ 18 years of age, literate and able to understand Urdu) culture confirmed MDR-TB patients enrolled for treatment at the study site from October 1, 2012 to September 30, 2013 were included in the study. Patients with a history of MDR-TB treatment, and physical and/or cognitive limitations that prevent them from being able to answer questions were excluded. Treatment protocol of MDR-TB patients enrolled in the current study has previously been reported elsewhere [11–12]

### Health Related Quality of Life Assessment Questionnaire

Short Form (SF-36) health survey has been used for numerous TB studies and showed acceptable reliability and validity [13–17]. The SF-36v2 comprised of eight scales that measure eight domains of HRQoL: physical functioning (PF, 10 items), role-physical (RP, four items), bodily pain (BP, two items), general health (GH, five items), vitality (VT, four items), social functioning (SF, two items) role-emotional (RE, three items) and mental health (MH, five items). These eight domains are grouped into two clusters known as physical component summary (PCS) and mental component summary (MCS). Physical functioning, RP and BP correlate strongly with PCS, whereas MH, RE, SF scales correlate with MCS. The remaining two scales VT and GH correlate moderately with both PCS and MCS [18]. Official Urdu version of SF36v2 standard form (one month recall period) used in this study was provided by QualityMetric, under the license number QM016042.

A pilot testing of SF-36v2 (Urdu translation) among 43 eligible MDR-TB patients other than those included in the final study at the study site showed a good internal consistency of all eight health domain scales. The reliability coefficients ranged from 0.72–0.83 and were well within the acceptable limits (Cronbach's α > 0.7) [19]. PCS had strong correlation with PF, RP, BP (r ≥ 0.5) and moderate correlation with GH (r = 0.49). Likewise, MCS had a strong correlation with ME, RE, SF (r ≥ 0.5), and moderate correlation with VT (r = 0.47).

### Data collection

During the study period, all eligible culture confirmed MDR-TB patients who agreed to participate in the study by giving a written consent were asked to self-complete SF-36v2 at three time points: i) at baseline visit ii) within two weeks of completion of 12 months of treatment and iii) at the end of treatment (>20 months of treatment). Enrolled subjects who did not participate at the second follow up were not asked to take the questionnaire on third follow up. Validated data collection form was used for collecting patients’ socio-demographic and clinical data. The study was approved by the Research and Ethics Committee of the Postgraduate Medical Institute, Peshawar, Pakistan.

### Scoring

For scoring questionnaires, QualityMetric Health Outcomes^™^ Scoring Software 4.5 was used. The standard norm based scoring (NBS) of eight health domains and summary components were calculated by using the standard scoring algorithms (US weights). This method is recommended by the developers of the instrument [18] and similar studies conducted elsewhere [17, 20–22]. A higher SF-36 score indicated better HRQoL outcome. During treatment, a change of ≥ 3 NBS points in summary component measures and health domain scales represented minimal important difference (MID) [18]. Scores ranging from 47 to 53 NBS points on component summary measures and health domain scales were considered equivalent to general population norms. A score of <47 NBS points was considered indicative of function impairment. An individual was considered at the risk of depression if he/she had an MCS score < 43 NBS points [17–18].

### Statistical analysis

Data was analyzed by SPSS 16. General Linear Model (GLM) repeated measures ANOVA was used to examine that i) whether there is a significant interaction effect and the subjects have a different pattern of change over the time, and ii) which independent variables were predictive of changes in summary scores. Upon observing statistically significant interaction, Bonferroni post hoc test was used for refining its interpretation. A p-value <0.05 was considered statistically significant. Cohen’s proposed commonly used guidelines were used for ranking the effect size (small, partial eta squared = 0.01; moderate, partial eta squared = 0.06; large, partial eta squared = 0.14) [23].

## Results

**Fig 1:**
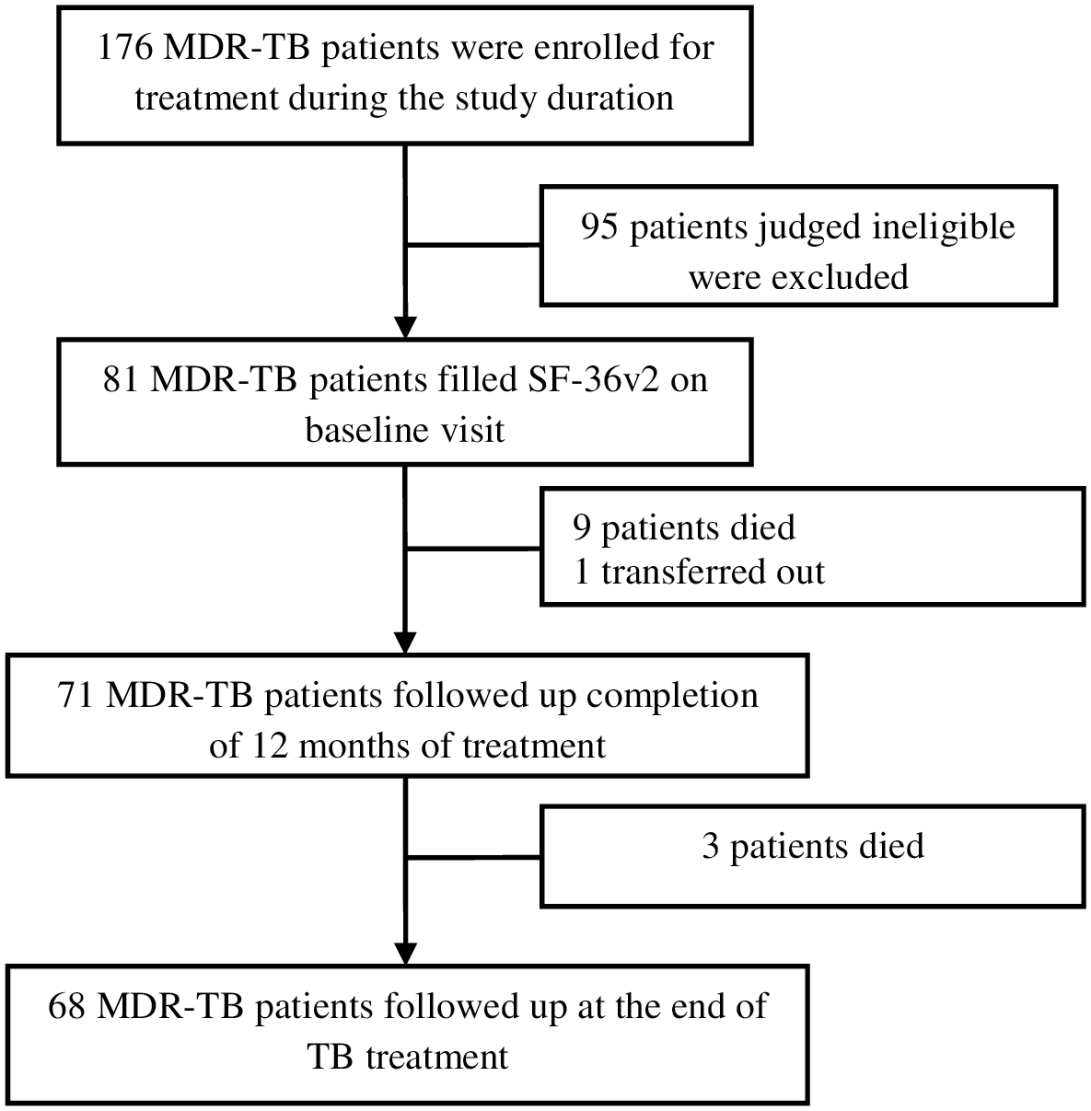
Flow diagram of patients screened, included and evaluated for impact of MDR-TB treatment on patients’ HRQoL.

During the study recruitment period, a total of 176 MDR-TB patients were enrolled for the treatment at the study site. Ninety five patients (54%) did not meet the eligibility criteria and were excluded. Eighty one patients were judged eligible and completed questionnaire at the baseline visit. Subsequently, 71 and 68 patients completed questionnaire on second and final follow up respectively (Fig 1). Baseline clinical and socio-demographic characteristics of the patients enrolled in the study are given in the table 1.

**Table 1:** Patients’ baseline socio-demographic and clinical characteristics (N = 81).

Variable	Mean + SD	No. (%)
**Gender**		
Female		20 (24.7)
Male		61 (75.3)
**Age (years)**	27.75+10.14	
< 40		62 (76.5)
> 40		19 (23.5)
**Weight**	47.90+8.30	
< 40		33 (40.7)
> 40		48 (59.3)
**Residence**		
Rural		51 (63.0)
Urban		30 (37.0)
**Marital status**		
Unmarried		45 (55.5)
Married		36 (44.5)
**Smoking**		
Non-smokers		69 (85.2)
Active + ex-smokers		12 (14.8)
**Education level**		
School		62 (76.5)
College		8 (9.9)
Graduation		11 (13.6)
**Employment**		
No		69 (85.2)
Yes		12 (14.8)
**Monthly family income (PKR)**		
<10000		31 (38.3)
10001–20000		30 (37)
>20000		20 (24.7)
**Previous TB treatment**		
No		8 (9.9)
Yes		73 (90.1)
**Length of sickness prior to the diagnosis of MDR-TB**		
<1 year		43 (53.1)
>1 year		38 (46.9)
**History of SLD use**		
No		76 (93.8)
Yes		5 (6.2)
**Co-morbidity**		
No		71 (86.7)
Yes		10 (12.3)
**Baseline smear grading**		
Negative		4 (4.9)
Scanty (1–9 AFB/100 HPF)		2 (2.5)
+1 (10–99 AFB/100 HPF)		24 (29.6)
+2 (1–9 AFB/HPF)		20 (24.7)
+3 (>9 AFB/HPF)		31 (38.3)
**Lung cavitations at baseline chest x-ray**		
No		52 (64.2)
Yes		29 (35.8)
**Number of resistant drugs**		
2–4		21 (25.9)
> 4		60 (74.1)
**Resistance to any SLD**		
No		38 (46.9)
Yes		43 (53.1)
**Baseline hemoglobin**		
Normal (male >13.5 gm/dl, female >12 gm/dl)		29 (35.8)
Below normal		52 (64.2)

AFB, acid fast bacilli; HPF, high power field; kg, kilogram; MDR-TB multidrug resistant TB, mg/dl milligram/deciliter; PKR, Pakistani Rupees; SLD, second-line anti-TB drugs, SD, standard deviation

Table 2 describes the NBS of eight health domain scales at different stages of MDR-TB treatment. At all three time points the mean scores for all health domains were <47 NBS points. Minimal important difference represented by a change of ≥3 NBS points was observed for all health domains between the first and final follow up, but no MID was observed for any health domain between the first and second follow up.

**Table 2:** SF-36v2 Health Domain Scores at different stages of treatment using Norm Based Scoring.

Scales	Mean scores ± SD			
	Baseline visit	Completion of 12 months of treatment	End of treatment	Mean change in score*
**Physical functioning**	37.5±4.7	38.1±4.5	41.1±5.1	3.6
**Role physical**	34.0±4.1	35.3±3.8	40.1±4.9	6.1
**Bodily pain**	39.7±4.2	39.4±4.5	43.7±6.3	4.0
**General health**	30.2±3.9	32.5±4.2	36.5±4.7	6.3
**Vitality**	39.0±5.3	39.6±5.2	42.4±4.8	3.4
**Social functioning**	32.8±5.9	34.9±6.0	38.8±6.5	6.0
**Role emotional**	30.1±4.9	32.1±5.2	37.6±5.3	7.5
**Mental health**	35.5±6.9	36.8±7.8	41.7±8.1	6.2

SD, standard deviation

*Mean change in health domain scale scores from the baseline visit to the end of treatment

Mean scores of component summary measures are presented in table 2. During the treatment, MID was observed for both PCS and MCS, but both scores remained <47 NBS points on all the three occasions. For both PCS and MCS scores, one-way repeated measure ANOVA revealed significant interaction effect for time (Table 3).

**Table 3:** Changes in mean component summary scores: one-way repeated measures ANOVA.

Summary component	Mean scores ± SD			Mean change in score*
	Baseline visit	Completion of 12 months of treatment	End of treatment	
**PCS**	38.2±4.7	38.6±4.4	42.2±5.2	4.0
**MCS**	33.7±7.0	35.5±6.9	40.0±6.9	6.3

PCS, physical component summary; MCS, mental component summary; SD, standard deviation

For PCS [Wilk’s Lambda = 0.405, F(2, 66) = 48.437, p<0.001, multivariate partial eta squared = 0.595]

For MCS [Wilk’s Lambda = 0.407, F (2, 66) = 47.996, p<0.001, multivariate partial eta squared = 0.593]

**Fig 2:**
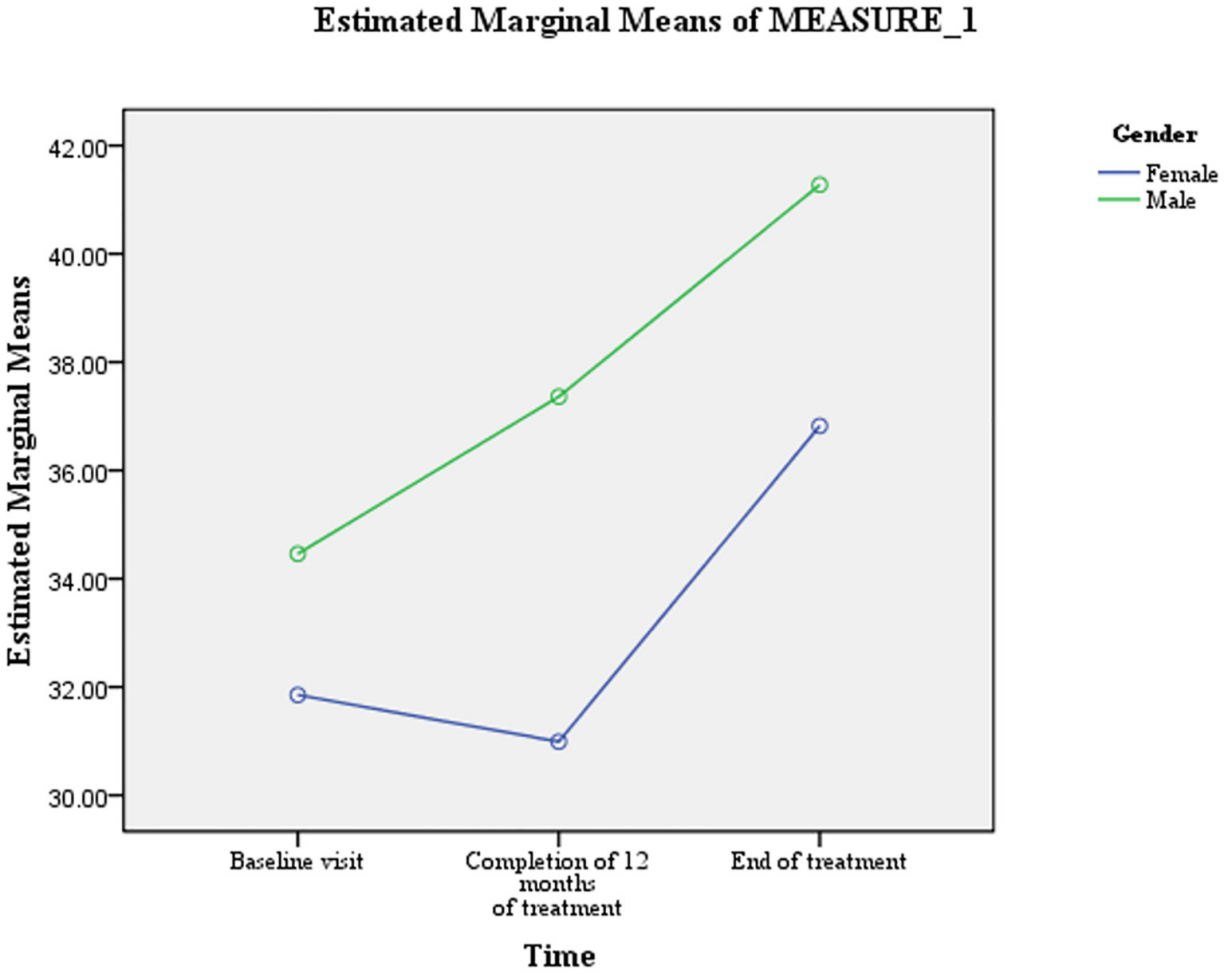
Patient’ gender: difference in the estimated marginal means of MCS scores.

Results of GLM repeated measure ANOVA presented in table 4 revealed that no independent variable interacted with time to predict trends in PCS scores. However, patients’ gender (F = 3.94, Df = 1.67, 110.48) and marital status (F = 3.60, Df = 1.76, 116.28) had statistically significant interaction with time to predict trends in MCS scores. For male patients, the differences in the mean MCS scores between all the time periods were statistically significant and characterized substantial. Whereas for female patients, the differences in mean MCS scores between the first and second time point were statistically non-significant and characterized minimal. Moreover, for female patients a slight decrease in mean MCS score was observed between the first and second time point (Fig 2).

**Table 4:** Test of within-subjects effects for the summary scores: GLM repeated measures ANOVA.

Source	Df	Error	F	p-value	Partial eta squared
Measure: physical component summary*					
Time*Male	1.485	98.001	1.017	0.346	0.015
Time*Age≥ 40 years	1.495	98.642	1.411	0.247	0.021
Time*Married	1.497	98.797	0.135	0.812	0.002
Time*Urban	1.496	98.719	0.138	0.810	0.002
Time*School level education	1.492	89.469	0.876	0.395	0.013
Time*Length of sickness prior to the diagnosis of MDR-TB ≥1 year	1.497	98.826	0.654	0.480	0.010
Time*Monthly family income>20000 PKR	1.489	98.261	0.867	0.395	0.013
Time*lung cavitation at baseline chest x-ray	1.463	96.528	1.719	0.191	0.025
Measure: mental component summary*					
Time*Male	1.674	110.488	3.942	**0.029**	0.056
Time*Age ≥ 40 years	1.714	113.150	0.508	0.575	0.008
Time*Married	1.762	116.285	3.602	**0.036**	0.052
Time*Urban	1.691	111.608	2.779	0.075	0.040
Time* School level education	1.706	112.564	0.774	0.445	0.012
Time*length of sickness prior to the diagnosis of MDR-TB ≥1 year	1.724	113.761	0.324	0.691	0.005
Time*Monthly family income>20000 PKR	1.734	114.458	1.049	0.345	0.016
Time*presence of lung cavities on chest x-ray	1.689	111.493	1.396	0.251	0.021

Df, degree of freedom; MDR-TB, multidrug resistant TB; PKR, Pakistani Rupees

*Greenhouse-Geisser values as sphericity cannot be assumed (p-value ≤0.001)

**Fig 3:**
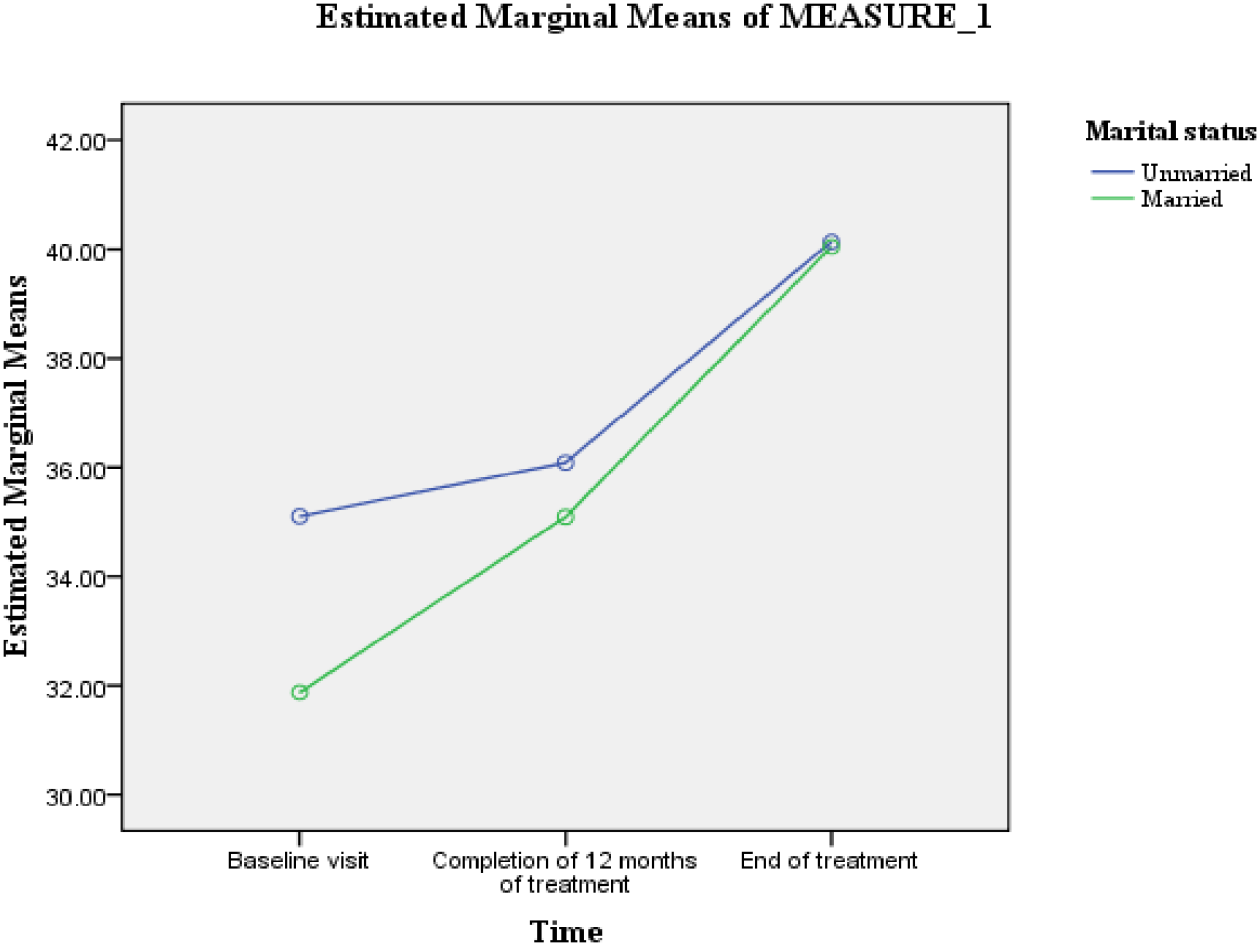
Marital status: difference in the estimated marginal means of MCS scores.

For married patients, the difference in MCS scores between all the time periods was statistically significant and characterized substantial. For unmarried patients, the difference in MCS scores between the first and second time point was statistically non-significant and characterized minimal (Fig 3).

**Fig 4:**
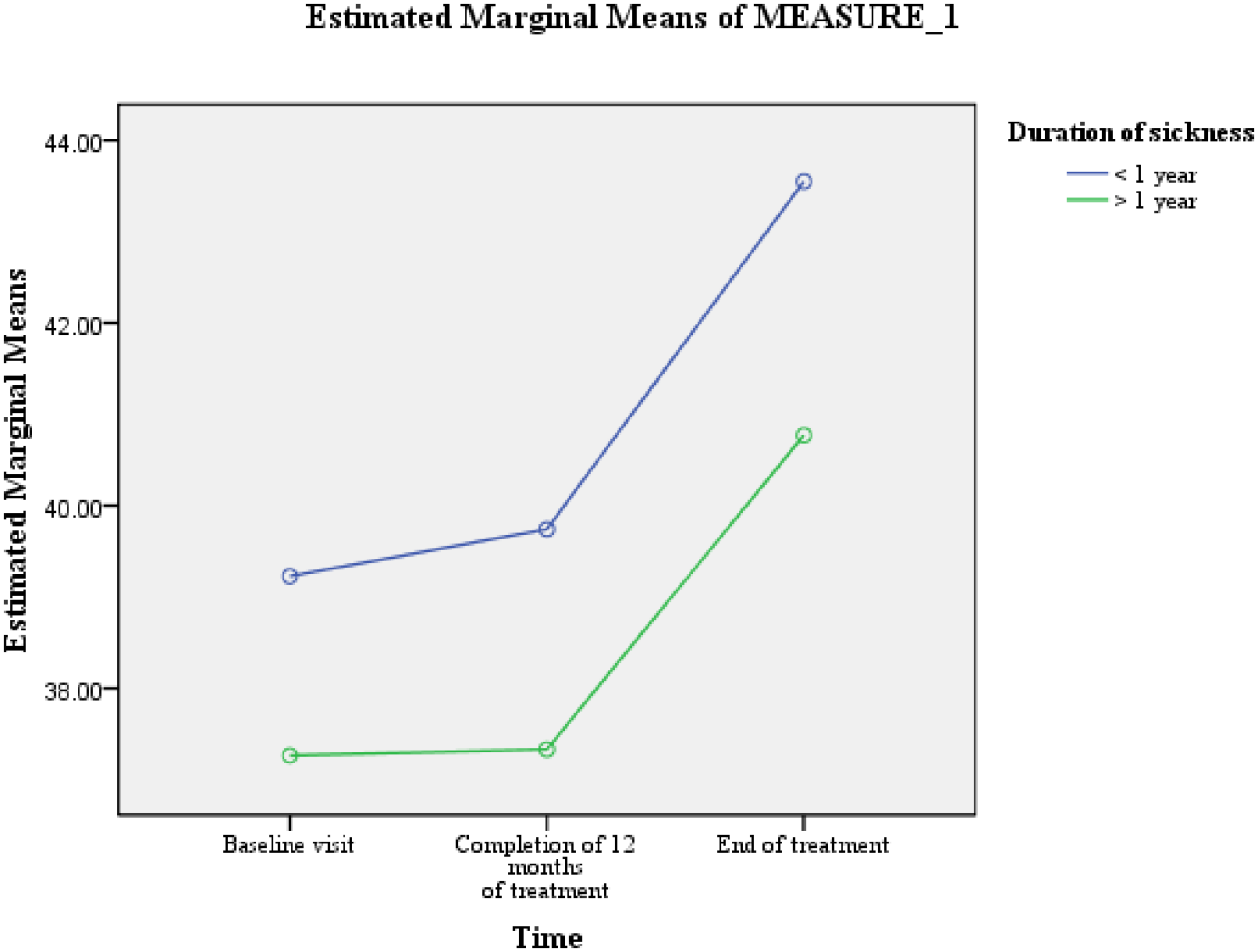
Length of sickness for ≥ 1 year prior to the diagnosis of MDR-TB: difference in the estimated marginal means of PCS scores.

**Fig 5:**
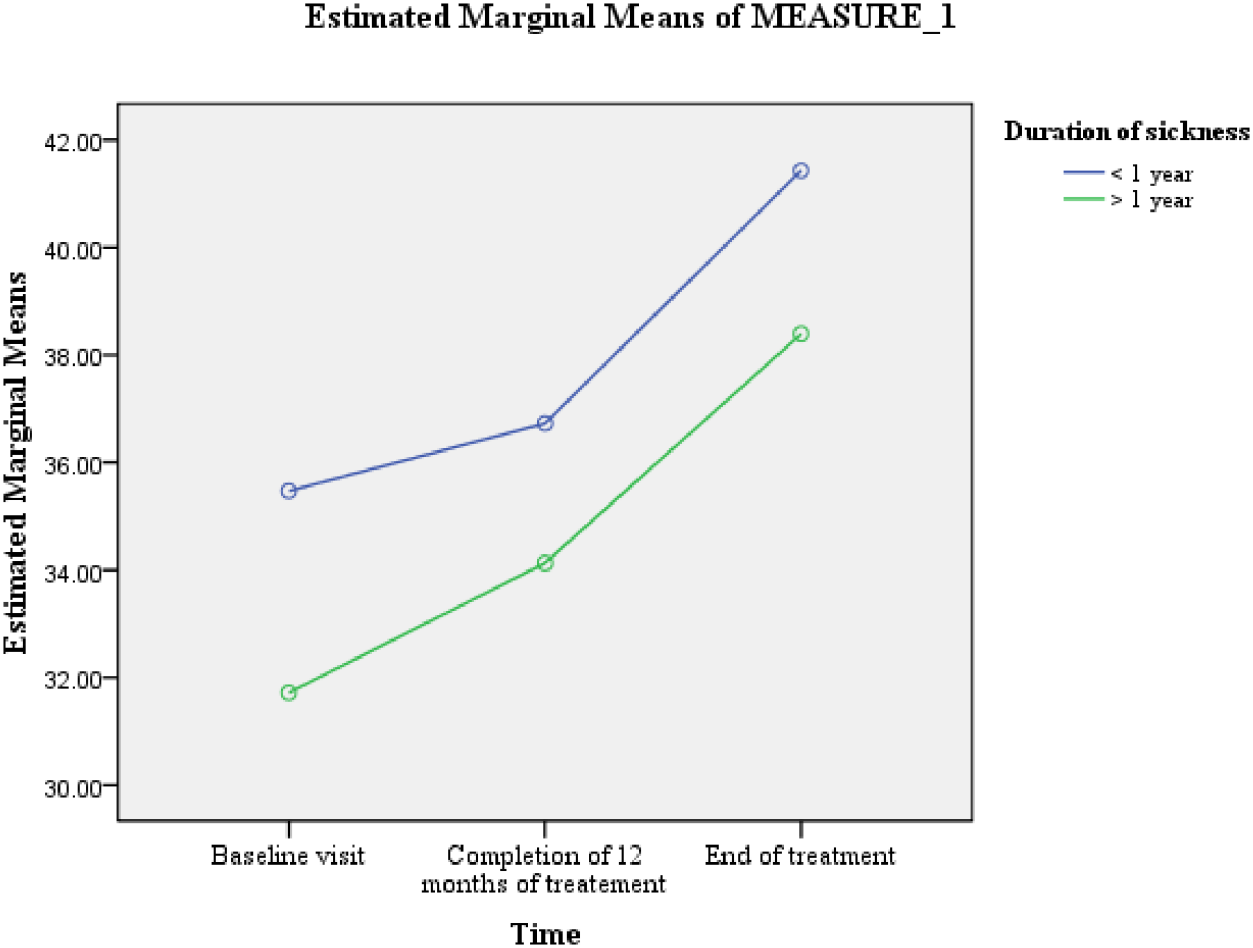
Length of sickness for ≥1 year prior to the diagnosis of MDR-TB: difference in the estimated marginal means of MCS scores.

Table 5 shows that the variable of length of sickness ≥1 year prior to the diagnosis of MDR-TB was predictive of difference in PCS scores (F = 4.988, Df = 1, 66) (Fig 4). Whereas, male gender (F = 5.638, Df = 1, 66) and length of sickness ≥1 year prior to the diagnosis of MDR-TB (F = 4.400, Df = 1, 66) were predictive of differences in MCS scores (Figs 2 & 5). As the difference between the groups at Time 2 and 3 were largely reflective of the difference observed at Time 1, it seems that the differences in the PCS and MCS scores were more likely to be attributed to the differences in the composition of the groups.

**Table 5:** Test of between-subjects effects for the summary scores: GLM repeated measures ANOVA.

Source	Df	Error	F	p-value	Partial eta squared
**Measure: physical component summary**					
Male	1	66	1.419	0.238	0.021
Age≥ 40 years	1	66	0.526	0.471	0.008
Married	1	66	0.257	0.614	0.004
Urban	1	66	0.192	0.663	0.003
School level education	1	66	0.778	0.381	0.012
Length of sickness prior to the diagnosis of MDR-TB ≥1 year	1	66	4.988	**0.020**	0.070
Monthly family income>20000 (PKR)	1	66	0.403	0.528	0.006
presence of lung cavities at baseline chest x-ray	1	66	1.018	0.317	0.015
**Measure: mental component summary**					
Male	1	66	5.638	**0.020**	0.078
Age≥ 40 years	1	66	3.312	0.073	0.047
Married	1	66	1.112	0.295	0.016
Urban	1	66	2.528	0.117	0.036
School level education	1	66	0.376	0.542	0.006
Length of sickness prior to diagnosis of MDR-TB ≥1 year	1	66	4.400	**0.040**	0.062
Monthly family income>20000 PKR	1	66	1.194	0.278	0.018
Lung cavitation at baseline chest x-ray	1	66	0.000	0.992	0.000

Df, degree of freedom; MDR-TB, multidrug resistant TB; PKR, Pakistani Rupees

## Discussion

To the best of our knowledge, this is the first longitudinal study which evaluated the impact of MDR-TB treatment on patients’ HRQoL. This study provides the much needed data about the impact of MDR-TB treatment on patients’ HRQoL. On initial evaluation, the study participants’ scores of <47 NBS on all eight health domain scales and summary measures suggested the severely deteriorated HRQoL of study participants. Similar poor HRQoL in patients with drug susceptible and MDR-TB has been reported by various studies conducted elsewhere [6–7, 9, 14, 17, 22, 24–25]. In the current study, due to the absence of control group of drug susceptible TB patients, we were unable to draw a definite conclusion that whether the severely deteriorated HRQoL at the baseline visit was due to MDR-TB or previous episode of TB. However, at the baseline visit, the comparatively worse HRQoL scores of the current study participants than the previously reported scores of drug susceptible TB patients [6–7, 9, 14, 17, 26–27] suggests the severe deteriorating effect of MDR-TB on patients’ HRQoL. On initial evaluation, we observed that role-emotional and general health of the study participants were the two most affected health domains. This indicated that patients had severe difficulties in carrying out their daily life activities due to emotional stress, and perceived their overall health as poor with the fear of further deterioration [17]. Comparative lower mean score on MCS than PCS was an indication of more mental distress and role limitation due to emotional problems rather than the physical problems. Similar lower scores on psychological health domain in patients with MDR and drug susceptible TB have been reported by studies conducted elsewhere [7, 17, 26].

Upon second evaluation, except the domain of bodily pain which worsened further, clincially non-signficant improvements were observed in all health domain scales and both component summary measures. The possible reason for increase in bodily pain between the two time points could be the painful injections and adverse effects associated with the drugs like pyrazinamide. Contrary to our finding, various studies among drug susceptible TB patients have reported MID at the completion of intensive phase of treatment [15, 17, 24, 28]. As, MDR-TB patients after initiating treatment have to adjust to the daily doses of multiple tablets and intramuscular injections of an extreme regimen associated with severe and painful side effects [5], this finding was not a surprising one, and shows how much challenging and uncomfortable was the initial phase of MDR-TB treatment. The participants of a qualitative study conducted elsewhere have described the intensive phase of MDR-TB treatment worse than the disease itself [5]. The uncomfortable intensive phase of MDR-TB treatment reflects well by the comparatively greater default rate during this phase of treatment [29].

As expected, at the end of TB treatment, the HRQoL scores of the study participants improved significantly in all eight health domains and both component summary measures. Similar positive impact of drug susceptible TB treatment has been reported by various studies conducted elsewhere [14, 16–17, 24]. Despite clinically significant improvements, the HRQoL scores of the study participants at the end of TB treatment were well below the US general population norms. Keeping in mind the chronic and destructive nature of MDR-TB, and prolonged therapy with toxic drugs for two years, one may expect high prevalence of residual impairment in HRQoL of these patients. Similar residual impairments of HRQoL among drug susceptible TB patients have been reported by a systematic review and individual studies conducted elsewhere [16–17, 27]. On final evaluation, the patients’ mean MCS score was comparatively lower than their PCS score. This finding was comparable with studies drug susceptible conducted elsewhere [24, 26].

Another noteworthy finding of the current study was the high prevalence of risk of depression among the study participants. On initial evaluation, more than 76% of the study participants were at the risk of depression (MCS <43 NBS points). Although, this proportion decreased along with MDR-TB treatment, but a notable proportion of patients (42%) were still at the risk of depression even at the end of treatment. We suppose that the previous multiple treatment failures, knowledge of suffering from a life-threatening form of TB, psychiatric effects associated with the likes of cycloserine, ethionamide and fluoroquinolones could be the possible reasons for high percentage of patients to be at the risk of depression in the current study [30–33]. In line with our findings, high prevalence of depression among MDR-TB patients have been reported by studies conducted elsewhere [15, 17, 30]. However, in contrast to 42% patients at the risk of depression at treatment completion in our study, prevalence of depression among drug susceptible TB patients at the end of TB treatment in a Malaysian study was only 23.5% [15]. As, the current study participants suffered from a more severe and chronic form of TB, were treated with drugs with known adverse effects of psychiatric disturbance, and lived a changed life style to accommodate the long course of MDR-TB painful therapy, one may expect comparatively high prevalence of depression among MDR-TB patients than drug susceptible TB patients at any stage of treatment.

We observed that the variable of length of sickness prior to the diagnosis of MDR-TB was predictive of differences in overall PCS and MCS scores. At all the three time points, patients who remained sick for ≥1 year prior to the diagnosis of MDR-TB significantly scored lower on both PCS and MCS. The well recognized physical deterioration of TB depends on the duration and severity of the disease [24], the longer the duration of sickness the more severe would be the physical deterioration [34–35]. As, the majority of patients who remained sick ≥ 1 year prior to the diagnosis of MDR-TB TB had received multiple episodes of TB treatment, their past experiences of long and complicated pathways of care before being diagnosed with MDR-TB, diagnostic and treatment fatigue, and possible lack of faith in treatment efficacy [36] could be the possible reasons for significantly lower mental health among them. The relatively longer period of hopelessness, social stigma, inadequate social support, sense of worthlessness, fear of disease and death could have contributed to the comparatively worse mental health among this group of patients. A positive association between length of sickness and depression among TB patients has previously been reported by studies conducted elsewhere [37–38]. Moreover, the direct relationship between the physical deterioration and lower mental health [39], and overproduction of interleukin-6 in chronic infections, which by facilitating the cascade of endocrine reactions results in depression could be other possible reasons [40] for significantly lower mental wellbeing in this group of patients. Poor HRQoL has been reported as a predictor of unsuccessful treatment outcomes among TB patients [41]. The significantly worse HRQoL among patients with longer duration of sickness prior to the diagnosis of MDR-TB stresses the need for early diagnosis of drug resistant TB. Among the current study participants, one of the reasons for longer duration of sickness prior to the diagnosis of MDR-TB was the general practice of treating Category-I failures with Category-II regimen rather than evaluating them for drug resistance. The effectiveness of Category-II regimen for Category-I failures has been doubtful especially in setting of a good DOTS, where most Category-I failures have MDR-TB [42–45]. Therefore, as per WHO guidelines, in an MDR-TB high burden country like Pakistan, Category-I failures should be tested for drug resistance rather than putting them on Category-II regimen [36, 46]. Access to rapid drug susceptibility testing (Xpert MTB/RIF assay) will reduce delay in the diagnosis of MDR-TB and will subsequently result in preventing further reduction in patients’ HRQoL, and better treatment outcomes. The other possible reason of prolonged duration of sickness with TB prior to the diagnosis of MDR-TB could be the “shopping around” by chest symptomatics before finally reaching the chest clinic and being diagnose with TB [47]. A study conducted among TB patients in seven countries of Eastern Mediterranean Region of WHO has reported that, nearly 50% of patients in Pakistan practiced self-medication and visited a mean of 5±3.6 health care providers before TB diagnosis was recorded [48]. Increasing public awareness about TB symptoms, hazards of self-medication and availability of free diagnostic and treatment at public health facilities, and building an effective collaboration between national tuberculosis control program (NTP) and private health sector can reduce delay in diagnosis of drug susceptible as well as resistant TB [47–48].

In the current study, patients’ gender was predictive of differences in overall MCS scores. Compared to male patients, mean MCS scores for female patients were significantly lower at all the three time points. Concurrent to our finding, significantly lower mental wellbeing among female TB patients has been reported by studies conducted elsewhere [22, 24, 49]. It has been widely reported that due to biological responses, self concepts and coping styles, women are two times more likely than men to develop depression [50–51], even when they are confronted with the same problems [50, 52]. Biologically females are more likely than males to have dysregualted hypothalamic-pitutary-adrenal axis (HPA) response to stress, which makes them more vulnerable to develop depression [53]. In developing countries like Pakistan, women lack social power and are marginalized socially and economically [54]. Suffering of socially and economically marginalized females from a chronic debilitating disease like MDR-TB could have affected their mental health comparatively more than their male counterparts. Patients’ gender in the present study also interacted with the time to predict changes in MCS scores. For male patients, the mean MCS score significantly increased between all the three time points. While for females, no significant change in mean MCS score was observed between the first two time points. Moreover, their mean MCS score decreased slightly between the first and second time point. This finding suggested that the mental health of the female MDR-TB patients worsened further during the first 12 months of treatment, and it stresses the need for greater care and emotional support to female MDR-TB patients during this phase of treatment. Although in the current study, patients’ marital status was not predictive of change in mean scores of component summary measures, but it interacted with the time to predict changes in mean MCS scores. As compared with unmarried patients, the mean MCS score of married patient increased substantially during the first 12 months of treatment. This difference could be a result of the supposed better care and emotional support provided by the spouses and children of married patients [36]. Moreover, marriage has been widely reported as a stronger protective factor for depressive symptoms [55–56].

### Conclusion

Despite the positive impact of MDR-TB treatment on patients’ HRQoL, the poor HRQoL of study participants even at the end of TB treatment warrants the urgent attention of NTP managers. It is suggested that HRQoL data of MDR-TB patients should be collected at various stages of MDR-TB treatment. This will provide an additional parameter to evaluate the efficacy of the treatment and effectiveness of the program, and will enable health care providers to take timely and appropriate actions to improve patients’ HRQoL. MDR-TB patients with the known risk factors for poor HRQoL need special attention. A large number of patients were at the risk of depression during MDR-TB treatment. In addition to monthly psychological counseling and material support in the form food ration and conveyance allowance, the provision of psychological support to MDR-TB patients through peer-to-peer and by support groups may enable patients to meet and socialize with other patients and give psychological support to each other. Inviting cured patients to support groups may provide emotional support to the patients on treatment.

### Limitations

Being a study from a single center in an MDR-TB high burden country, the findings of the present study should be interpreted with the major limitation of small number of patients enrolled. However, at the time of study initiation, the study site was the only center in Khyber Pukhtoonkhwa (one of the four provinces of Pakistan) where MDR-TB patients from all over the province, tribal and northern areas, and nearby Punjab were referred for treatment. As, the current study included patients from a widely distributed geographical area, and MDR-TB patients at all PMDT units in the country are treated with uniform protocols, we believe that the current study findings could reflect the impact of MDR-TB treatment on patients’ HRQoL at other PMDT units. Nevertheless, a multicenter study with a large sample size and drug susceptible TB controls is needed to confirm the findings of the current study. As, we used the self administered version of SF-36v2 health survey, and majority of the patients enrolled at the study site for treatment were illiterate, they were unable to participate in the study. Because of limited number of smokers and patients with co-morbidities in the current cohort, we were unable to include these two variables in to GLM repeated measure ANOVA to evaluate their impact on patients’ HRQoL.
